# Supplementary material for: BMI-adjusted adipose tissue volumes exhibit depot-specific and divergent associations with cardiometabolic diseases
Source: Nat Commun. 2023 Jan 17;14:266. doi: 10.1038/s41467-022-35704-5 (PMC9844175; doi:10.1038/s41467-022-35704-5)
Supplement: Supplementary file 1 — Supplementary Information [file 41467_2022_35704_MOESM1_ESM.pdf]

Supplementary Appendix

BMI-adjusted adipose tissue volumes exhibit depot-specific and divergent associations with cardiometabolic diseases

Agrawal and Klarqvist et al.

Supplementary Methods

Supplementary Figure 1 Grad-CAM in male participants with high absolute error

Supplementary Figure 2 Grad-CAM in female participants with high absolute error

Supplementary Figure 3 Grad-CAM in male participants in held out datasets for VAT, ASAT, GFAT

Supplementary Figure 4 Grad-CAM in female participants in held out datasets for VAT, ASAT, GFAT

Supplementary Figure 5 Density plots of VATadjBMI, ASATadjBMI, and GFATadjBMI

Supplementary Figure 6 Correlogram of fat depots adjusted-for-BMI and anthropometric measurements

Supplementary Figure 7 Linear versus spline fit for fat depot volumes versus BMI

Supplementary Figure 8 Fat depot specific effects on type 2 diabetes across age, sex, and BMI subgroups

Supplementary Figure 9 Fat depot specific effects on coronary artery disease across age, sex, and BMI subgroups

Supplementary Methods

Raw MRI imaging data in the UK Biobank

We studied body magnetic resonance imaging (MRI) data from 43,531 participants of the UK BIobank study. As previously described, the UK Biobank is an observational study that enrolled over 500,000 between the ages of 40 and 69 years between 2006 and 2010.[1] In an ongoing effort, up to 100,000 participants from four regions in the UK will be asked to return for comprehensive imaging – at the time of this study, 43,531 participants had been imaged and had MRI imaging data available for download.[2]

Participants in the MRI imaging substudy were scanned using Siemens Aera 1.5-T MRI scanners from the neck to the knees using the Dixon method, an MRI sequence that can be used to isolate fat from water signal.[3] Image acquisition from the neck to the knees was comprised of 6 contiguous stages with varying numbers of axial slices: stage 1 covered the neck with 64 slices, stages 2-4 covered the torso with 44 slices each, stage 5 covered the upper legs with 72 slices, and stage 6 covered the lower legs with 64 slices.[2]

The output of this procedure is a set of four MRI sequences for each participant: in-phase and out-of-phase ("phase" in reference to water and fat molecule precessions), and fat-only and water-only (obtained by adding and subtracting in-phase and out-of-phase, respectively).

Quality control of MRI imaging data

Starting with raw imaging data for 43,531 participants, 389 participants had either incomplete acquisitions (e.g. missing a stage), or faulty or corrupted data (e.g. length of pixel data does not match the metadata records). After exclusion of these 389 participants, we performed quality control of the remaining 43,142 sets of two-dimensional projections and noted 7 classes of imaging artifacts that occurred:

- Fat/water swaps involving a complete stage, N = 412
- Knee(s) missing and/or metal artifacts in the body, N = 257
- Field-of-view errors caused by a misaligned individual in either superior-inferior direction such that the head or chin was partly or fully visible or the clavicle was not fully visible, N = 397
- Participant was too large to be completely captured in the scanner view, N = 107
- Instance swaps with self-contained areas of fat/water swaps within a stage, N = 325
- Liver swaps where fat/water instance swaps are restricted to the superior part of the liver, N = 1,289
- Technically unusable because of unexpected observations such as stage-wide duplications or stages in the incorrect order, blank stages, or extremely noisy acquisitions, N = 323

In total, 3,499 (8.0%) of individuals were excluded by our quality control pipeline – consistent with a prior estimate of imaging artifact in UK Biobank body MRI data – resulting in 40,032 participants who were carried forward into downstream analysis.[4]

Fat depots previously quantified in a subset of UK Biobank participants

Among UK Biobank participants who underwent MRI imaging study, a subset had visceral adipose tissue (VAT) volume, abdominal subcutaneous adipose tissue (ASAT) volume, and total adipose tissue between the bottom of the thigh muscles to the top of vertebrae T9 (TAT) volume quantified and made available via the UK Biobank portal to the broader research community.[4–9] VAT (field 22407, "volume of the adipose tissue within the abdominal cavity, excluding adipose tissue outside the abdominal skeletal muscles and adipose tissue and lipids within and posterior of the spine and posterior of the back muscles") was available in 9,978 participants, ASAT (field 22408, "volume of the subcutaneous adipose tissue in the abdomen from the top of the femoral head to the top of the thoracic vertebrae T9") was available in 9,979, and TAT (field 22415, "total volume of adipose tissue, measured by MRI, between the bottom of the thigh muscles to the top of vertebrae T9") was available in 8,524. Based on these definitions, we additionally computed gluteofemoral adipose tissue (GFAT) volume:

GFAT = TAT (between top of T9 and bottom of thigh muscles) - VAT - ASAT

Given that the vast majority of adipose tissue between the top of vertebrae T9 and the top of the femoral head is accounted for by VAT or ASAT, GFAT was defined as total adipose tissue between the top of the femoral head and the bottom of the thigh muscles. Among 33,989 of the 40,032 (85%) participants with both body MRI and DEXA data available, we verified that MRI-derived GFAT and DEXA-derived gynoid fat – a related quantity – had good agreement (R^2 = 91-92%).

Simplifying three-dimensional MRI images to two-dimensional mean projections

Each of the 6 contiguous stages that make up an individual's MRI in the UK Biobank are acquired at slightly different resolutions, and so a pre-processing step was required prior to subsequent analysis. Resolutions ranged from 2.232 x 2.232 x 4.5 mm^3 (stages 2-4) to 2.232 x 2.232 x 3.0 mm^3 (stage 1). We resampled each series to the highest available resolution (voxel = 2.232 x 2.232 x 3.0 mm^3), enabling a merged single three-dimensional volume that included all stages.

We next evaluated the computational burden associated with training machine learning models on 3D MRI data. We noted that three-dimensional MRI data for >40,000 individuals represented a substantial data burden with almost 58 million axial slices across all participants, corresponding to >18 terabytes of imaging data – a level of complexity that limits computational feasibility for training deep learning models.

To simplify the machine learning model inputs, we transformed MRI images for each participant into two-dimensional projections of the coronal and sagittal anatomical planes – hypothesizing that this input would prove adequate for accurate fat depot volume quantification. Similar reduced representations of MRI imaging data were used in a recent study with excellent model performance.[10] Coronal and sagittal two-dimensional projections were generated by computing the mean intensity projection in each orientation. For example, a given pixel on a coronal two-dimensional projection represents the mean intensity across all pixels making up a line oriented in the anterior-posterior direction perpendicular to the coronal plane. This procedure was done for each MRI sequence for each participant.

Convolutional neural networks (CNNs) to quantify fat depots

Among the 40,032 individuals who remained after quality control, 9,040 participants had VAT quantified, 9,041 participants had ASAT quantified, and 7,754 participants had GFAT quantified and made available via previous studies.[4–9] For each fat depot volume, participants were split into 80% for model training and 20% for model validation (**Supplementary Data 1-2**). We used 5-fold cross-validation within the 80% model training data to estimate error.

For each of these three fat depot volumes, a CNN was trained on a pair of fat phase and water phase images, where each image was composed of (a) a coronal two-dimensional projection and (b) a sagittal two-dimensional projection of the body MRI to predict each fat depot volume. Each CNN was developed with the DenseNet-121 architecture pre-trained on ImageNet as the base model.[11,12] The last dense block output was flattened using a global average pooling layer and then fed into three fully connected layers of size 64, 256, and 1, with the last layer having no activation function (linear mapping). All other activation functions use the ReLU non-linearity. All models were trained using the Adam optimizer with a learning rate set to a cosine decay policy decaying from 0.001 to 0 over 100 epochs, a shrinkage loss function using the hyperparameters a = 10.0 and c = 0.2, and a batch size of 32.[13,14]

For all training data, the following augmentations (random permutations of the training images) were applied: random shifts in the *XY*-plane by up to ±16 pixels, rotations by up to ±5 degrees around its center axis, and the coronal view horizontally flipped with a probability of 50%. Each view (coronal and sagittal) were separately pre-normalized by its *z*-score (0 mean, standard deviation of 1), followed by joint normalization following concatenation side-by-side.

Performance of each CNN developed here is shown in **Supplementary Data 3**.

Code availability

Code used to ingest whole-body Dixon MRI images from UK Biobank participants is made available at https://github.com/broadinstitute/ml4h/tree/master/ml4h/applications/ingest under an open-source BSD license.

Supplementary Figure 1 Grad-CAM in male participants with high absolute error

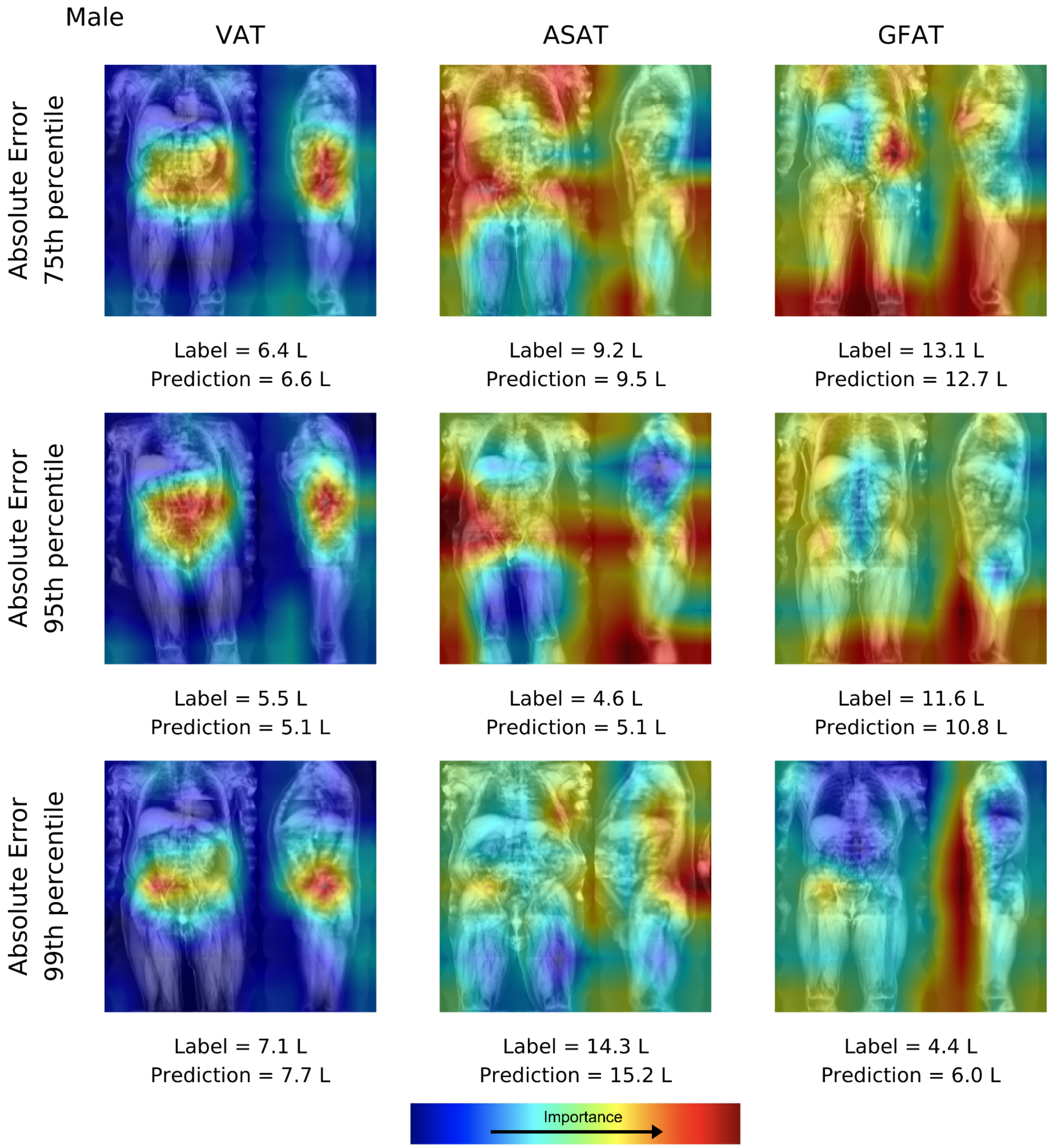

Results from saliency mapping using Grad-CAM are shown for nine male participants among the held out datasets at the 75th, 95th, or 99th percentiles of absolute error of prediction for either VAT, ASAT, or GFAT.

Supplementary Figure 2 Grad-CAM in female participants with high absolute error

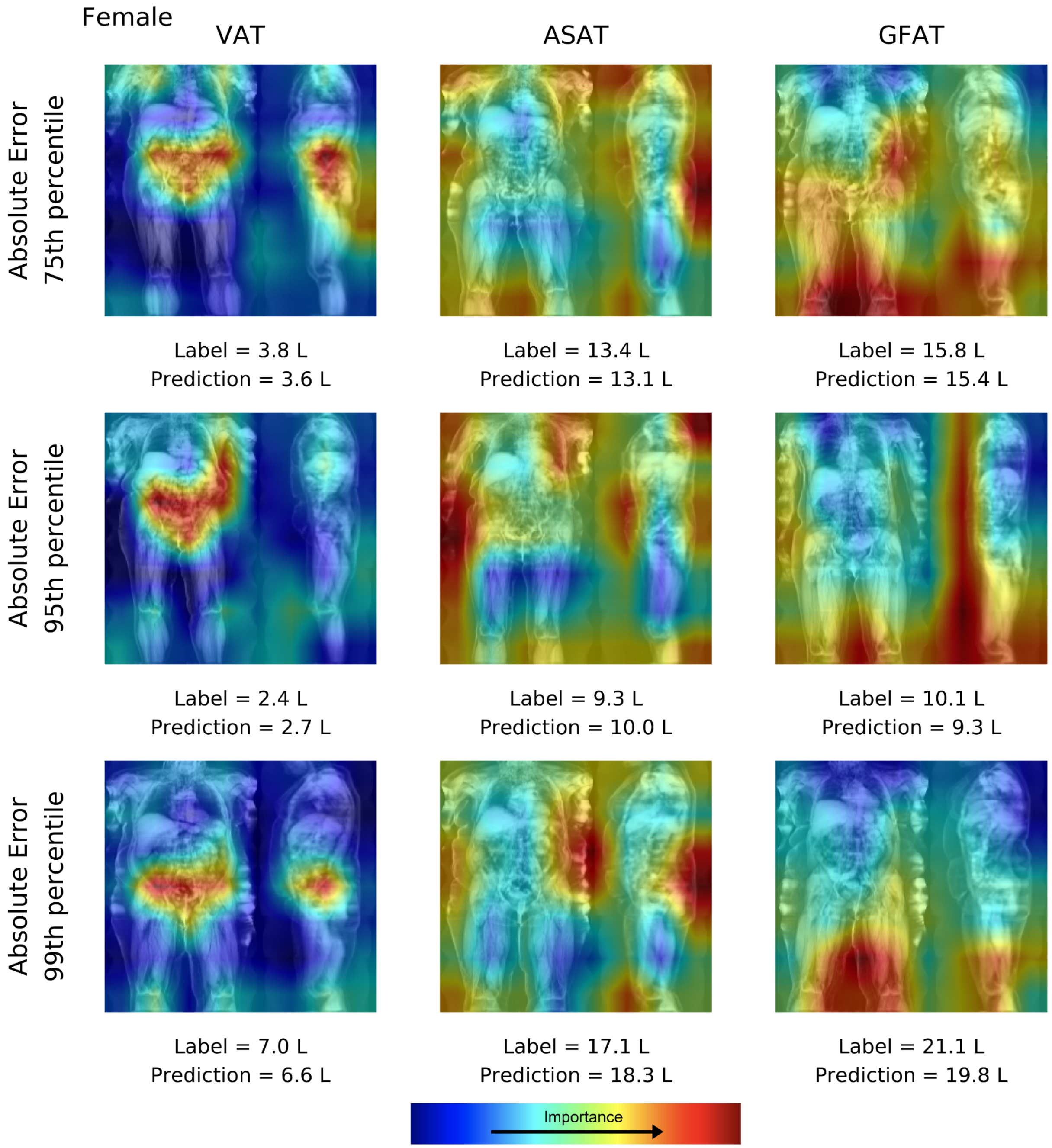

Results from saliency mapping using Grad-CAM are shown for nine female participants among the held out datasets at the 75th, 95th, or 99th percentiles of absolute error of prediction for either VAT, ASAT, or GFAT.

Supplementary Figure 3 Grad-CAM in male participants in held out datasets for VAT, ASAT, GFAT

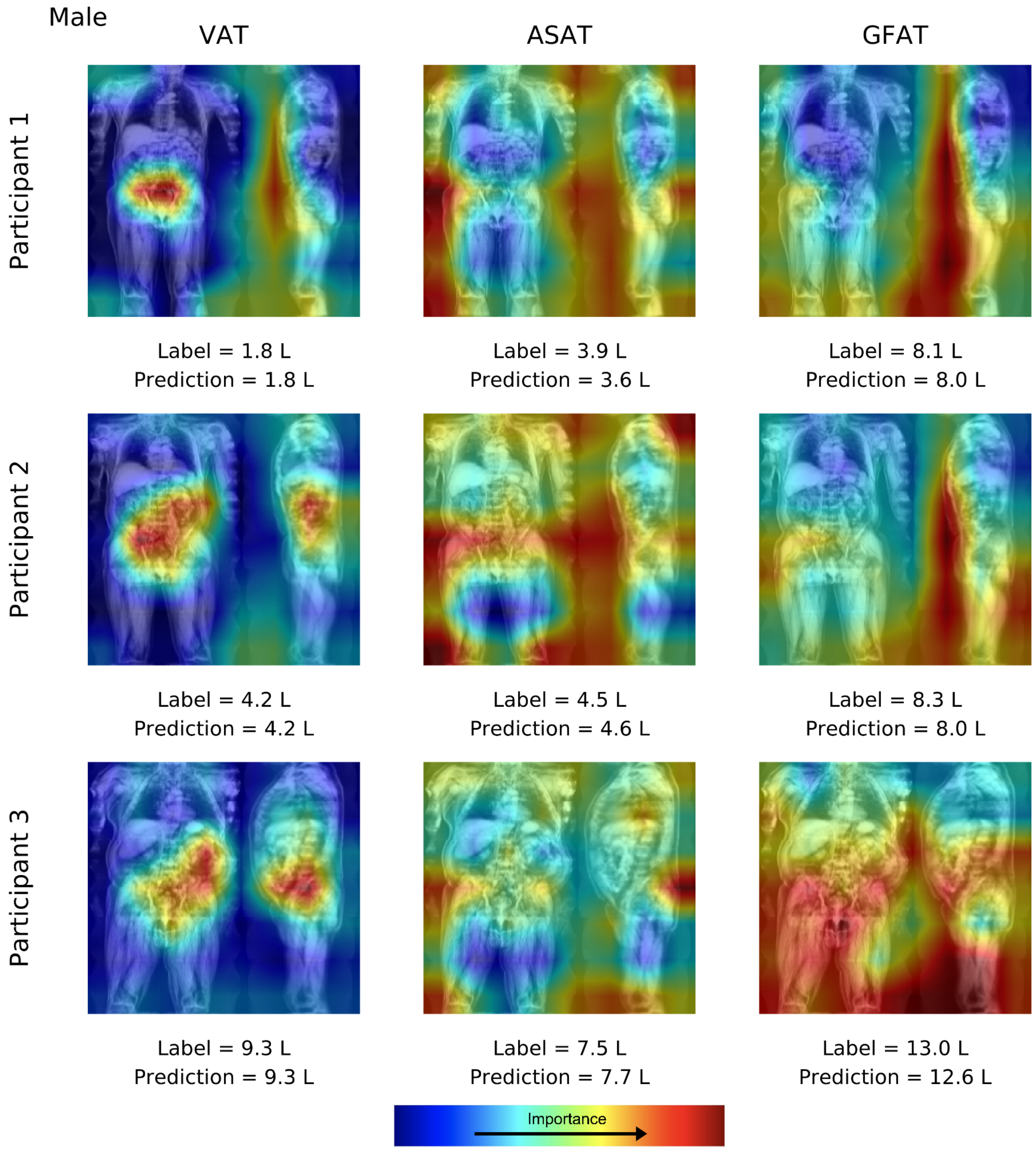

Results from saliency mapping using Grad-CAM are shown for three male participants who were in the held out datasets for all three of the VAT, ASAT, and GFAT models.

Supplementary Figure 4 Grad-CAM in female participants in held out datasets for VAT, ASAT, GFAT

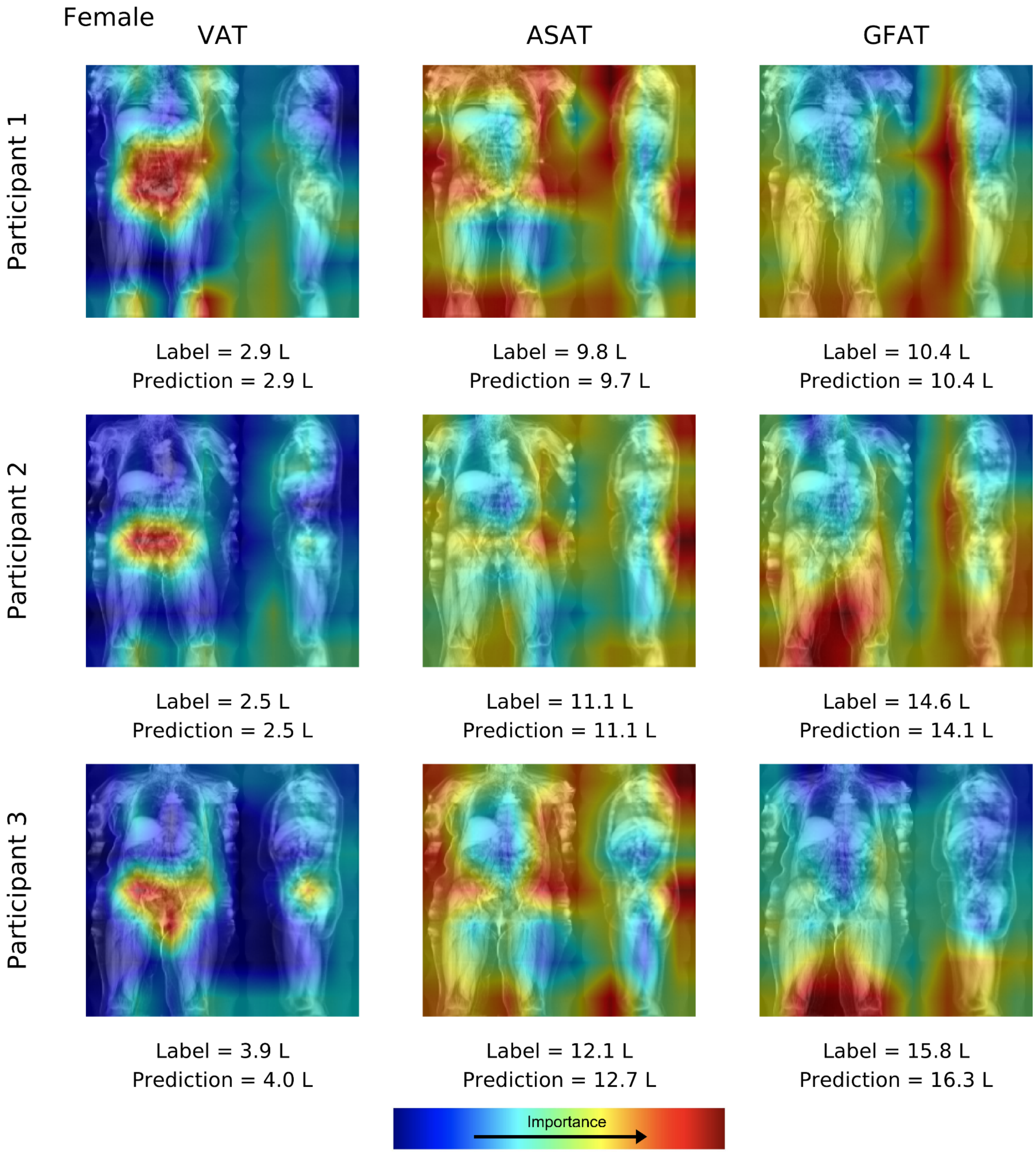

Results from saliency mapping using Grad-CAM are shown for three female participants who were in the held out datasets for all three of the VAT, ASAT, and GFAT models.

Supplementary Figure 5 Density plots of VATadjBMI, ASATadjBMI, and GFATadjBMI

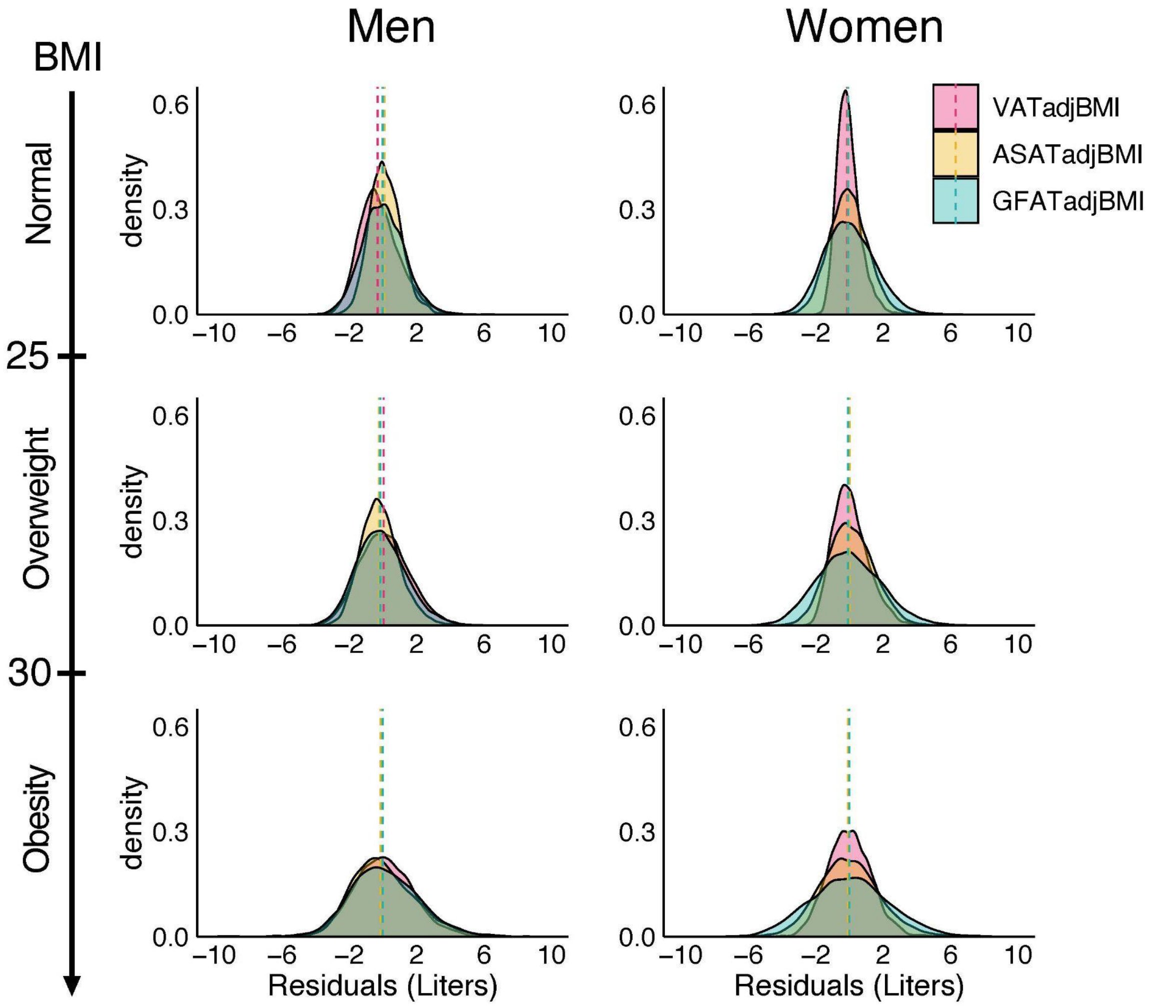

Sex- and BMI-group specific density plots for visceral adipose tissue volume adjusted for body-mass index (BMI), (VATadjBMI), abdominal subcutaneous adipose tissue volume adjusted for BMI (ASATadjBMI), and gluteofemoral adipose tissue volume adjusted for BMI (GFATadjBMI).

Supplementary Figure 6 Correlogram of fat depots adjusted-for-BMI and anthropometric measurements

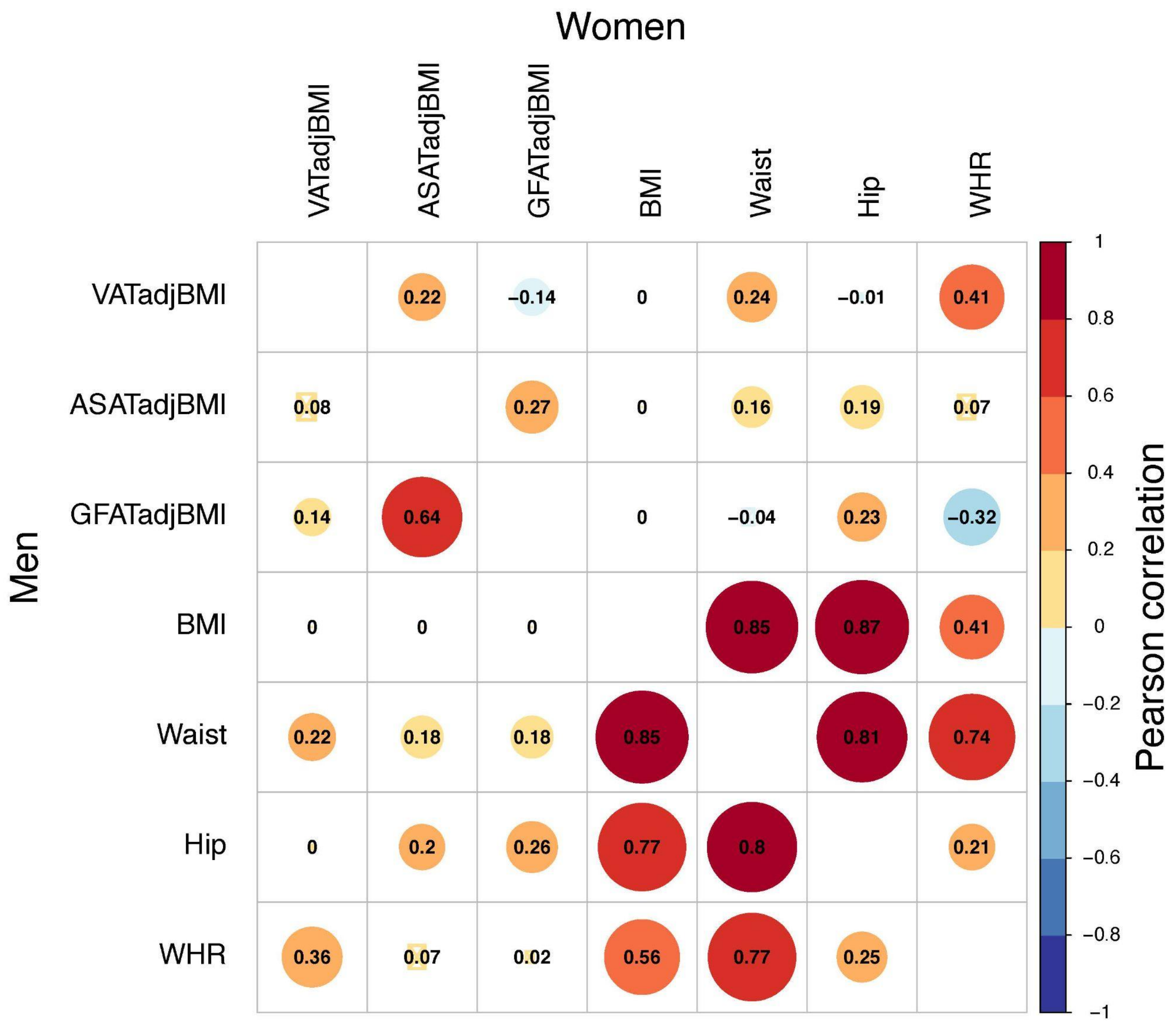

Sex-stratified correlation plots between visceral adipose tissue volume adjusted for BMI (VATadjBMI), abdominal subcutaneous adipose tissue adjusted for BMI (ASATadjBMI), gluteofemoral adipose tissue volume adjusted for BMI (GFATadjBMI) and four anthropometric measures: body mass index (BMI), waist circumference (Waist), hip circumference (Hip), and waist-hip ratio (WHR).

Supplementary Figure 7 Linear versus spline fit for fat depot volumes versus BMI

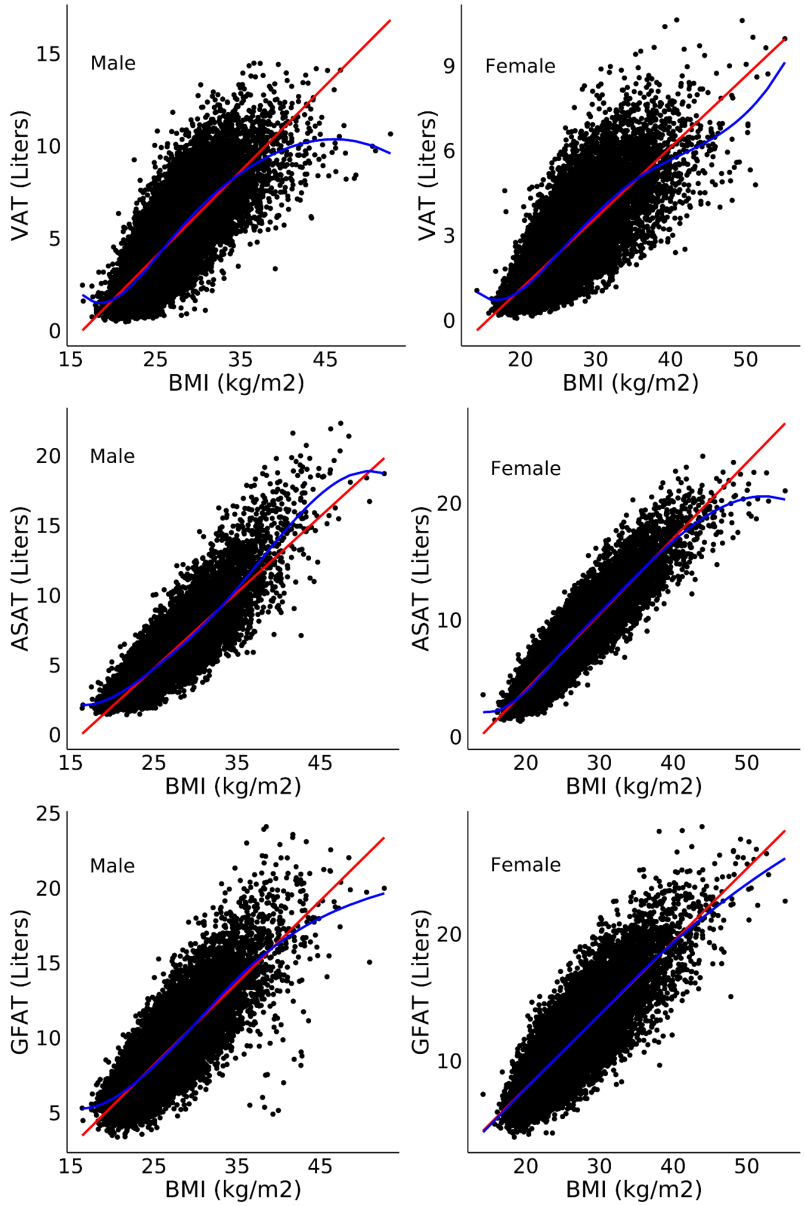

Sex-specific linear fits of each fat depot volume versus BMI are plotted in red. Sex-specific spline fits using a B-spline basis with knots at BMI = 25, 30, and 35 kg/m2 are plotted in blue. Because of good agreement between these two fits for most participants in the cohort (99% of participants lie between BMI 18 and 42 kg/m2), we proceeded with a linear adjustment. Pearson correlation between residuals obtained from each fit are shown in Supplementary Data 9.

Supplementary Figure 8 Fat depot specific effects on type 2 diabetes across age, sex, and BMI subgroups

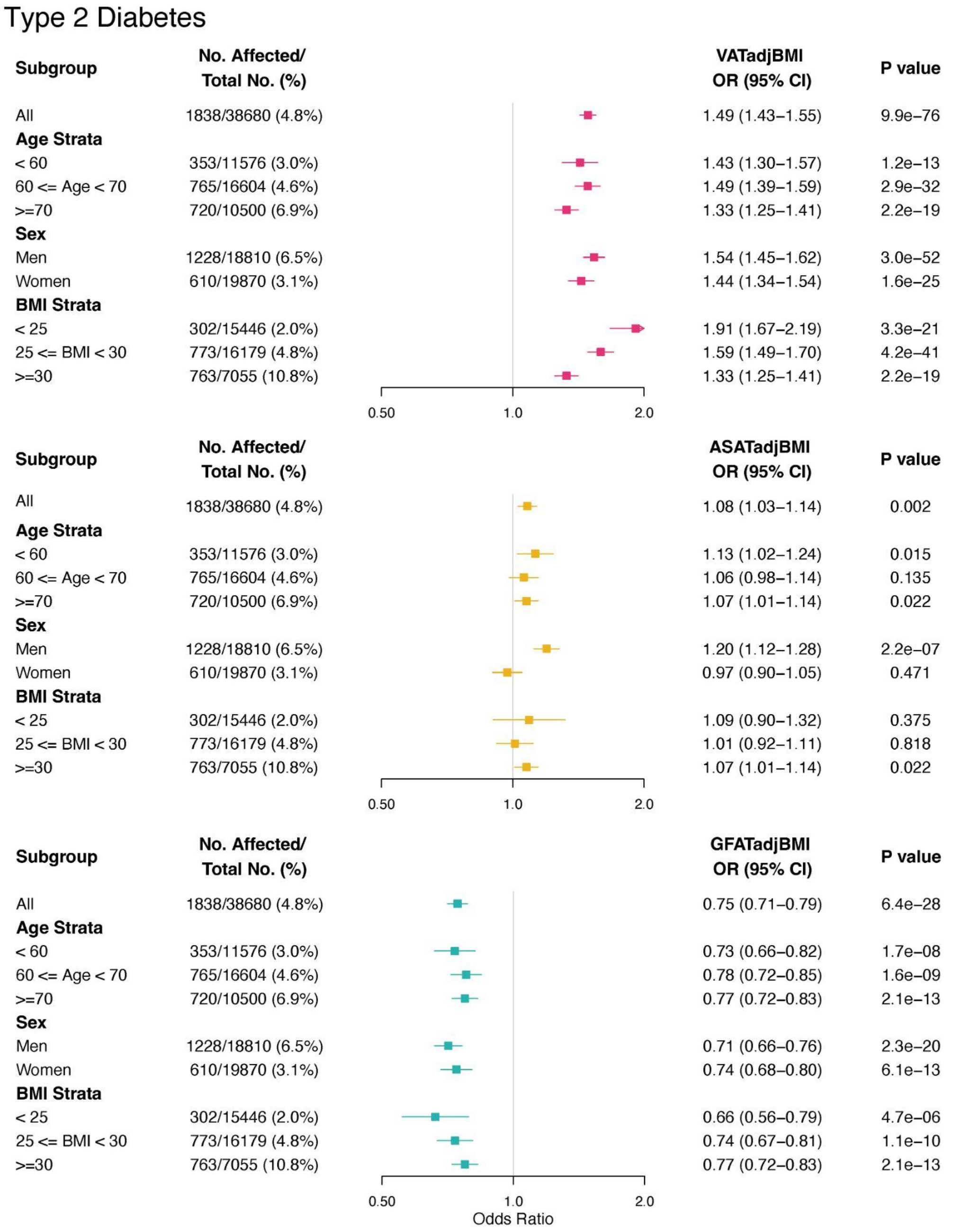

Odds ratios per standard deviation with 95% confidence intervals are shown for prevalent type 2 diabetes in demographic subgroups. Sample sizes for each model are shown as the denominator in the second column. P-values correspond to two-sided tests for the indicated independent variable in an adjusted logistic regression. Logistic regression models were adjusted for age, sex (except in sex subgroup analyses), BMI, the other two fat depots (e.g. ASATadjBMI and GFATadjBMI for VATadjBMI), and MRI imaging center. Source data are provided as a Source Data file.

Supplementary Figure 9 Fat depot specific effects on coronary artery disease across age, sex, and BMI subgroups

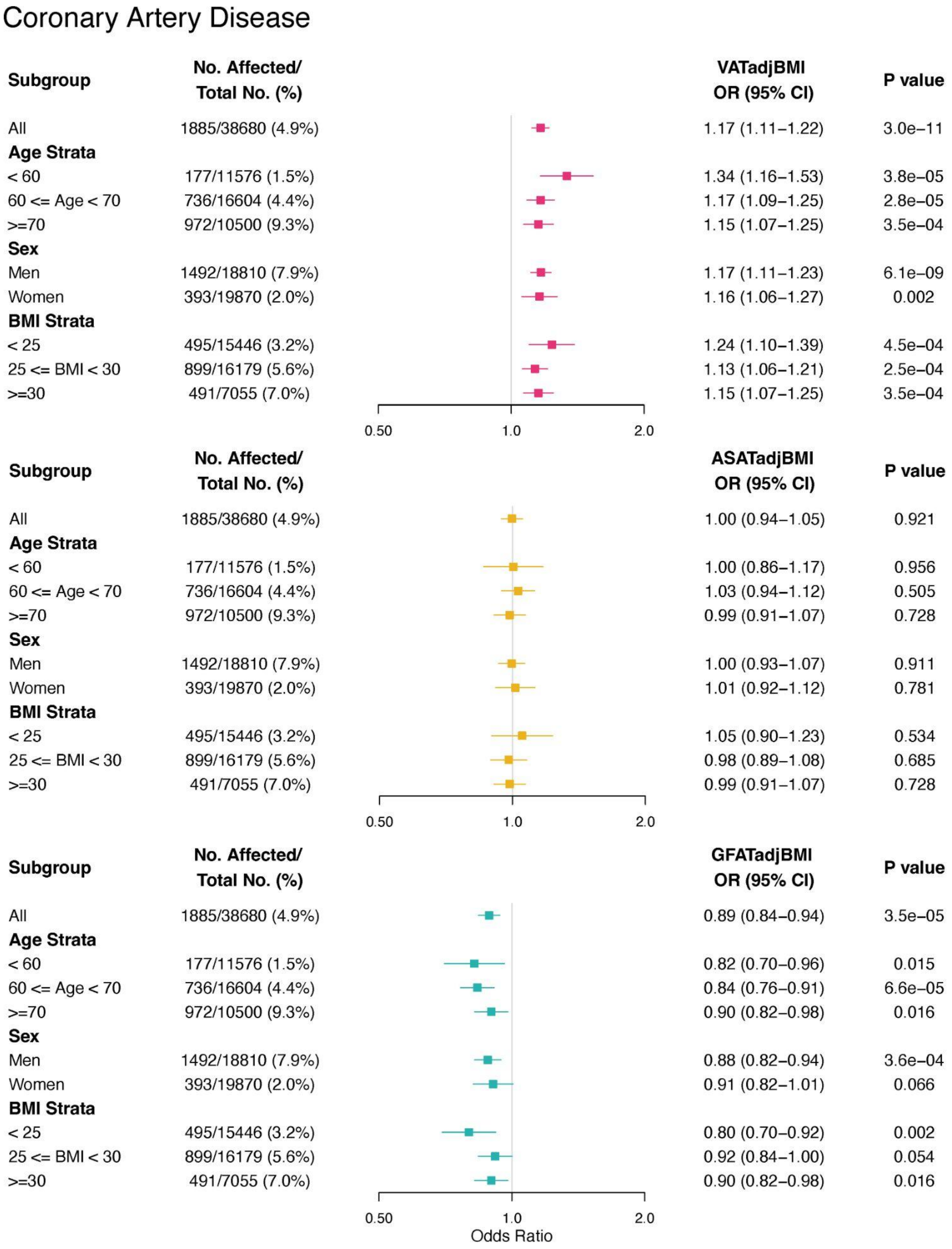

Odds ratios per standard deviation with 95% confidence intervals are shown for prevalent type 2 diabetes in demographic subgroups. Sample sizes for each model are shown as the denominator in the second column. P-values correspond to two-sided tests for the indicated independent variable in an adjusted logistic regression. Logistic regression models were adjusted for age, sex (except in sex subgroup analyses), BMI, the other two fat depots (e.g. ASATadjBMI and GFATadjBMI for VATadjBMI), and MRI imaging center. Source data are provided as a Source Data file.

References

1. Sudlow C, Gallacher J, Allen N, Beral V, Burton P, Danesh J, Downey P, Elliott P, Green J, Landray M, Liu B, Matthews P, Ong G, Pell J, Silman A, Young A, Sprosen T, Peakman T, Collins R. UK biobank: an open access resource for identifying the causes of a wide range of complex diseases of middle and old age. *PLoS Med* 2015;**12**:e1001779.
2. Littlejohns TJ, Holliday J, Gibson LM, Garratt S, Oesingmann N, Alfaro-Almagro F, Bell JD, Boultwood C, Collins R, Conroy MC, Crabtree N, Doherty N, Frangi AF, Harvey NC, Leeson P, Miller KL, Neubauer S, Petersen SE, Sellors J, Sheard S, Smith SM, Sudlow CLM, Matthews PM, Allen NE. The UK Biobank imaging enhancement of 100,000 participants: rationale, data collection, management and future directions. *Nat Commun* 2020;**11**:2624.
3. Dixon WT. Simple proton spectroscopic imaging. *Radiology* 1984;**153**:189–194.
4. West J, Leinhard OD, Romu T, Collins R, Garratt S, Bell JD, Borga M, Thomas L. Feasibility of MR-Based Body Composition Analysis in Large Scale Population Studies. *PLOS ONE* 2016;**11**:e0163332.
5. Leinhard OD, Johansson A, Rydell J, Smedby O, Nystrom F, Lundberg P, Borga M. Quantitative abdominal fat estimation using MRI. *2008 19th International Conference on Pattern Recognition*. 2008. p1–4.
6. Borga M, Thomas EL, Romu T, Rosander J, Fitzpatrick J, Dahlqvist Leinhard O, Bell JD. Validation of a fast method for quantification of intra-abdominal and subcutaneous adipose tissue for large-scale human studies. *NMR Biomed* 2015;**28**:1747–1753.
7. Borga M, West J, Bell JD, Harvey NC, Romu T, Heymsfield SB, Dahlqvist Leinhard O. Advanced body composition assessment: from body mass index to body composition profiling. *J Investig Med Off Publ Am Fed Clin Res* 2018;**66**:1–9.
8. Linge J, Borga M, West J, Tuthill T, Miller MR, Dumitriu A, Thomas EL, Romu T, Tunón P, Bell JD, Dahlqvist Leinhard O. Body Composition Profiling in the UK Biobank Imaging Study. *Obes Silver Spring Md* 2018;**26**:1785–1795.
9. Linge J, Whitcher B, Borga M, Dahlqvist Leinhard O. Sub-phenotyping Metabolic Disorders Using Body Composition: An Individualized, Nonparametric Approach Utilizing Large Data Sets. *Obes Silver Spring Md* 2019;**27**:1190–1199.
10. Langner T, Strand R, Ahlström H, Kullberg J. Large-scale biometry with interpretable neural network regression on UK Biobank body MRI. *Sci Rep* 2020;**10**:17752.
11. Huang G, Liu Z, Van Der Maaten L, Weinberger KQ. Densely Connected Convolutional Networks. *2017 IEEE Conference on Computer Vision and Pattern Recognition (CVPR)*. 2017. p2261–2269.
12. Deng J, Dong W, Socher R, Li L, Kai Li, Li Fei-Fei. ImageNet: A large-scale hierarchical image database. *2009 IEEE Conference on Computer Vision and Pattern Recognition*. 2009. p248–255.
13. Kingma DP, Ba J. Adam: A Method for Stochastic Optimization. *ArXiv14126980 Cs* 2017.
14. Lu X, Ma C, Ni B, Yang X, Reid I, Yang M-H. Deep Regression Tracking with Shrinkage Loss. 2018. p353–369.
