# Supplementary material for: BMI-adjusted adipose tissue volumes exhibit depot-specific and divergent associations with cardiometabolic diseases
Source: Nat Commun. 2023 Jan 17;14:266. doi: 10.1038/s41467-022-35704-5 (PMC9844175; doi:10.1038/s41467-022-35704-5)
Supplement: Supplementary file 3 — Description of Additional Supplementary Files [file 41467_2022_35704_MOESM3_ESM.pdf]

Title: Supplementary Data 1
Description: Baseline characteristics stratified by truth label status

Title: Supplementary Data 2
Description: Baseline characteristics stratified by development and holdout sets for fat depot CNNs

Title: Supplementary Data 3
Description: Convolutional neural network performance for adipose tissue volumes

Title: Supplementary Data 4
Description: Convolutional neural network performance in subgroups

Title: Supplementary Data 5
Description: Correlation between anthropometric traits across self-reported ethnicity groups

Title: Supplementary Data 6
Description: Type 2 diabetes definition

Title: Supplementary Data 7
Description: Coronary artery disease definition

Title: Supplementary Data 8
Description: Cardiometabolic disease associations with visceral, abdominal subcutaneous, and gluteofemoral adipose tissue volumes

Title: Supplementary Data 9
Description: Correlation between BMI residuals from a linear fit versus spline fit

Title: Supplementary Data 10
Description: Subgroup associations with prevalent type 2 diabetes

Title: Supplementary Data 11
Description: Subgroup associations with prevalent coronary artery disease

Title: Supplementary Data 12
Description: Interaction of BMI with BMI-adjusted fat depots

Title: Supplementary Data 13
Description: Association with prevalent cardiometabolic diseases: sensitivity analyses

Title: Suppementary Data 14
Description: Standardized prevalence of type 2 diabetes across quintiles of VATadjBMI, ASATadjBMI, and GFATadjBMI

Title: Supplementary Data 15
Description: Standardized prevalence of coronary artery disease across quintiles of VATadjBMI, ASATadjBMI, and GFATadjBMI

Title: Supplementary Data 16
Description: Definitions of ideal diet and physical activity

Title: Supplementary Data 17
Description: Association of healthy diet and physical activity with fat depots
