# Supplementary material for: BMI-adjusted adipose tissue volumes exhibit depot-specific and divergent associations with cardiometabolic diseases
Source: Nat Commun. 2023 Jan 17;14:266. doi: 10.1038/s41467-022-35704-5 (PMC9844175; doi:10.1038/s41467-022-35704-5)
Supplement: Supplementary file 21 — Reporting Summary [file 41467_2022_35704_MOESM21_ESM.pdf]

nature portfolio

Corresponding author(s): Saaket Agrawal, Amit V. Khera

Last updated by author(s): Dec 3, 2022

Reporting Summary

Nature Portfolio wishes to improve the reproducibility of the work that we publish. This form provides structure for consistency and transparency in reporting. For further information on Nature Portfolio policies, see our Editorial Policies and the Editorial Policy Checklist.

Statistics

For all statistical analyses, confirm that the following items are present in the figure legend, table legend, main text, or Methods section.

n/a	Confirmed	
☐	☒	The exact sample size (n) for each experimental group/condition, given as a discrete number and unit of measurement
☐	☒	A statement on whether measurements were taken from distinct samples or whether the same sample was measured repeatedly
☐	☒	The statistical test(s) used AND whether they are one- or two-sided<br>*Only common tests should be described solely by name; describe more complex techniques in the Methods section.*
☐	☒	A description of all covariates tested
☐	☒	A description of any assumptions or corrections, such as tests of normality and adjustment for multiple comparisons
☐	☒	A full description of the statistical parameters including central tendency (e.g. means) or other basic estimates (e.g. regression coefficient) AND variation (e.g. standard deviation) or associated estimates of uncertainty (e.g. confidence intervals)
☐	☒	For null hypothesis testing, the test statistic (e.g. F, t, r) with confidence intervals, effect sizes, degrees of freedom and P value noted<br>*Give P values as exact values whenever suitable.*
☒	☐	For Bayesian analysis, information on the choice of priors and Markov chain Monte Carlo settings
☒	☐	For hierarchical and complex designs, identification of the appropriate level for tests and full reporting of outcomes
☐	☒	Estimates of effect sizes (e.g. Cohen's d, Pearson's r), indicating how they were calculated

Our web collection on statistics for biologists contains articles on many of the points above.

Software and code

Policy information about availability of computer code

Data collection	All data were provided by the UK Biobank. No software was used for data collection.
Data analysis	Code used to ingest whole-body Dixon MRI images from UK Biobank participants is made available at the following Github repository under an open-source BSD license: https://github.com/broadinstitute/ml4h/tree/master/ml4h/applications/ingest. After QC, each convolutional neural network was developed with the publicly available DenseNet-121 architecture pre-trained on ImageNet as the base model. All downstream analyses were performed with the use of R software,v ersion 3.6.0(R Project for Statistical Computing).

For manuscripts utilizing custom algorithms or software that are central to the research but not yet described in published literature, software must be made available to editors and reviewers. We strongly encourage code deposition in a community repository (e.g. GitHub). See the Nature Portfolio guidelines for submitting code & software for further information.

Data

Policy information about availability of data

All manuscripts must include a data availability statement. This statement should provide the following information, where applicable:

- Accession codes, unique identifiers, or web links for publicly available datasets
- A description of any restrictions on data availability
- For clinical datasets or third party data, please ensure that the statement adheres to our policy

All data were provided by the UK Biobank; no software was used for data collection. This research has been conducted using the UK Biobank Resource under

Application Number #7089. The raw UK Biobank data - including the anthropometric data reported here - are made available to researchers from universities and other research institutions with research inquiries following IRB and UK Biobank approval. All other relevant results are available in the supplemental information.

Human research participants

Policy information about studies involving human research participants and Sex and Gender in Research.

Reporting on sex and gender	51% of participants in the study were female, as reported in Table 1. Results are presented stratified by sex throughout the manuscript in light of the significant sex dimorphism of body fat distribution.
Population characteristics	As described in Table 1, mean age was 64.5 years, 51% were female, and 97% were white. Mean body mass index for both males (27.1) and females (26.1) was in the overweight category.
Recruitment	The UK Biobank is an observational study that enrolled over 500,000 individuals between the ages of 40 and 69 years between 2006 and 2010 (via mailer recruitment), of whom 43,521 underwent MRI imaging between 2014 and 2020.
Ethics oversight	Mass General Brigham institutional review board; UK Biobank application #7089.

Note that full information on the approval of the study protocol must also be provided in the manuscript.

Field-specific reporting

Please select the one below that is the best fit for your research. If you are not sure, read the appropriate sections before making your selection.

☒ Life sciences ☐ Behavioural & social sciences ☐ Ecological, evolutionary & environmental sciences

For a reference copy of the document with all sections, see nature.com/documents/nr-reporting-summary-flat.pdf

Life sciences study design

All studies must disclose on these points even when the disclosure is negative.

Sample size	43,531 individuals with MRI images were available in the UK Biobank. After imaging QC described in the Supplementary Information, 40,032 remaining images were studied in this manuscript. Sample size was determined by MRI images available for download from the UK Biobank at the time of this study. Study power was adequate to determine statistically significant associations with type 2 diabetes and coronary artery disease. No power calculations were done.
Data exclusions	Imaging level QC was done on the basis of several criteria including fat/water swaps, individuals being misaligned, and individuals being too large for the scanner -- these are fully described in the Supplementary Information.
Replication	Performance of each of the three CNNs built to predict visceral, abdominal subcutaneous, and gluteofemoral fat volumes was benchmarked in held out validation sets that the CNNs were blinded to during model development. For all three models, performance in the held out validation set was R2 >= 97%. Validity of the regression approach in this manuscript was further supported by saliency mapping demonstrating anatomically consistent regions highlighting for VAT, ASAT, and GFAT, respectively.
Randomization	Not applicable to this prospective observational cohort study. All disease association models included covariates to age, sex, body mass index, and MRI imaging center.
Blinding	Investigator blinding was not applicable to this prospective observational cohort study. CNNs were blinded to held out validation sets during model development.

Reporting for specific materials, systems and methods

We require information from authors about some types of materials, experimental systems and methods used in many studies. Here, indicate whether each material, system or method listed is relevant to your study. If you are not sure if a list item applies to your research, read the appropriate section before selecting a response.

Materials & experimental systems

n/a	Involved in the study
☒	☐ Antibodies
☒	☐ Eukaryotic cell lines
☒	☐ Palaeontology and archaeology
☒	☐ Animals and other organisms
☒	☐ Clinical data
☒	☐ Dual use research of concern

Methods

n/a	Involved in the study
☒	☐ ChIP-seq
☒	☐ Flow cytometry
☒	☐ MRI-based neuroimaging
